# Cervical Lymph Node Metastasis Patterns and Diagnostic Accuracy of Preoperative Staging in Oral Squamous Cell Carcinoma

**DOI:** 10.3390/cancers18111851

**Published:** 2026-06-05

**Authors:** Michael-Tobias Neuhaus, Giulia Weniger, Efthymios Papazacharias, Fabian Fenske, Philipp Jehn, Fritjof Lentge, Philippe Korn, Nils-Claudius Gellrich, Rüdiger Zimmerer

**Affiliations:** 1 Department of Oral and Maxillofacial Surgery, Hannover Medical School, 30625 Hannover, Germany; 2 Private Practice, 04209 Leipzig, Germany; 3 Department of Oral & Maxillofacial and Facial Plastic Surgery, University Hospital Essen, 45239 Essen, Germany

**Keywords:** oral squamous cell carcinoma, cervical lymph node metastasis, elective neck dissection, occult metastasis, preoperative staging

## Abstract

**Background**: Reliable assessment of cervical lymph node metastases remains a key challenge in the management of oral squamous cell carcinoma (OSCC). While elective ipsilateral neck dissection (ND) is widely accepted, the benefit of contralateral ND and the influence of tumor site on metastatic risk remain incompletely defined. This study aimed to evaluate patterns of lymphatic metastases, the diagnostic accuracy of preoperative staging, and the therapeutic relevance of ipsilateral and contralateral ND. **Methods**: A retrospective single-center cohort study was conducted including 287 patients with histologically confirmed OSCC treated between 2013 and 2019. Patterns of lymph node metastases were analyzed with respect to tumor localization and clinicopathological factors. Multivariate binary logistic regression was performed to identify predictors of cervical lymph node metastases. The diagnostic accuracy of preoperative staging was evaluated using histopathological findings as the reference standard. **Results**: Tumor localization and histopathological grading significantly influenced the occurrence of lymph node metastases. OSCC of the maxilla demonstrated a significantly lower observed rate of cervical and occult metastases compared with other tumor sites. Occult metastases were detected in 16.9% of primary tumor cases, with only two contralateral occult metastases observed. The calculated number needed to treat (NNT) was 6 for ipsilateral elective ND and 74 for contralateral elective ND. Preoperative staging showed limited diagnostic accuracy, with a negative predictive value of 0.83 and a positive predictive value of 0.65. **Conclusions**: Elective ipsilateral ND remains an essential component in the surgical management of OSCC due to the considerable rate of occult metastases and the limited reliability of preoperative staging. In contrast, the benefit of contralateral elective ND appears limited in patients without midline-crossing tumors. Maxillary OSCC and well-differentiated tumors demonstrated a significantly lower metastatic risk, supporting a more individualized risk-adapted approach to neck dissection in selected cases.

## Simple Summary

This retrospective single-center study evaluated patterns of cervical lymph node metastasis, the diagnostic accuracy of preoperative staging, and the clinical value of ipsilateral and contralateral neck dissection (ND) in 287 patients with oral squamous cell carcinoma (OSCC). Associations between tumor localization, histopathological characteristics, and metastatic spread were analyzed. Tumor site and histopathological grading were identified as significant predictors of cervical lymph node metastases. Maxillary OSCC showed substantially lower rates of both cervical and occult metastases compared with other oral cavity subsites. Occult metastases were detected in 16.9% of primary tumor cases, whereas only two contralateral occult metastases were observed. The calculated number needed to treat (NNT) was 6 for elective ipsilateral ND, indicating a clear therapeutic benefit, compared with 74 for elective contralateral ND, suggesting limited clinical value. Preoperative staging demonstrated only moderate diagnostic performance, with a negative predictive value of 0.83 and a positive predictive value of 0.65. These findings support elective ipsilateral ND as an essential component of OSCC management due to the considerable prevalence of occult metastases and the limited reliability of preoperative staging. In contrast, elective contralateral ND appears to provide little benefit in patients without midline-crossing tumors. The lower metastatic risk observed in maxillary and well-differentiated tumors supports a more individualized, risk-adapted approach to neck dissection in selected patients.

## 1. Background

Oral squamous cell carcinoma (OSCC) is one of the most common cancers worldwide, with a diagnosis of 250 000 patients and 63 500 deaths reported in Europe in 2012 [1]. The 5-year overall survival is approximately 50% [2]. Standard treatments include surgical tumor resection, neck dissection (ND), and reconstruction of the tissue defects with subsequent facultative radiation and chemotherapy [3]. Primary radiation and chemotherapy are considered effective alternatives to surgical resection in advanced-stage disease.

After years of scientific discourse on the purpose of elective ND compared with therapeutic ND, recent studies have indicated a noticeable advantage of elective ND in terms of overall patient survival, cancer-free survival, and quality of life [4,5,6,7]. Elective ND includes ipsilateral cervical lymph node levels 1–3. Moreover, the German treatment guidelines (AWMF) also recommend elective ND [3]. However, little is known about the influence of specific tumor sites, stages, and other prognostic factors on lymph node invasion. Better knowledge of those tumor properties could allow for a more precise patient-specific treatment planning, reducing morbidity and increasing disease-free and overall survival.

Pretherapeutic staging involves clinical examination, computed tomography (CT) of the head and neck, ultrasound of the neck and abdomen, and chest radiography. However, extended staging using computed tomography of the chest and abdomen is recommended only for advanced-stage disease. Despite major improvements in diagnostic imaging, the sensitivity and specificity of radiographic staging examinations (CT and magnetic resonance imaging) for lymph node invasion remain limited, ranging between 0.72–0.77 and 0.72–0.81, respectively [8]. These values are comparable to the reported rate of preoperatively undetected occult cervical lymph node metastases of approximately 25%. Owing to this considerable rate of undetected lymph node invasion, elective ND remains standard practice in the management of OSCC. Although current German guidelines indicate a lower tendency for lymph node metastasis in maxillary OSCC, robust clinical evidence supporting omission of elective ND in selected cases remains limited.

Most previous studies have focused primarily on ipsilateral elective ND. In contrast, a large proportion of patients in the present cohort underwent bilateral elective ND, providing a unique opportunity to systematically assess both ipsilateral and contralateral lymph node metastasis patterns and to evaluate the diagnostic performance of preoperative staging under conditions comparable to surgical open staging.

Therefore, the aim of this retrospective study was to systematically analyze lymphatic metastasis patterns in patients with OSCC undergoing ND, with particular emphasis on the following objectives:

1. To identify clinicopathological factors associated with cervical lymph node metastasis.
2. To evaluate site-specific patterns of lymph node metastasis across different oral cavity subsites.
3. To assess the occurrence and clinical relevance of contralateral lymph node metastases in patients undergoing bilateral ND.
4. To determine the diagnostic performance of preoperative staging in detecting cervical lymph node metastases.

## 2. Methods

This retrospective single-center cohort study included patients treated at the Department of Oral and Maxillofacial Surgery, Hannover Medical School, Hannover, Germany. The institutional database and clinical records were screened for all consecutive patients who underwent surgical treatment including neck dissection (ND) between January 2013 and December 2019.

Patient identification was performed using International Classification of Diseases (ICD-10) codes for head and neck malignancies (C00–C14) and corresponding procedural codes (OPS 5-403) for neck dissection. Only patients with histologically confirmed oral squamous cell carcinoma (OSCC) were included to ensure a homogeneous study cohort. Patients with primary tumors, secondary tumors, and recurrent disease were eligible for inclusion if complete clinical and histopathological records were available.

Patient data were pseudonymized prior to analysis. The study was conducted in accordance with the Declaration of Helsinki and approved by the local ethics review committee (No. 9257_BO_K_2020).

Patients were eligible for inclusion if they met the following criteria:

• Age ≥ 18 years.
• Histologically confirmed oral squamous cell carcinoma (OSCC), including carcinomas of the oral aspect of the lip.
• Surgical treatment including neck dissection (ND).
• Availability of complete clinical, radiological, and histopathological records.
• Written informed consent for the use of clinical data for research purposes.

### 2.1. Data Collection and Statistical Analysis

Clinical and pathological data were extracted from electronic medical records and institutional databases. The following variables were recorded: patient demographics, risk factors (tobacco and alcohol consumption), tumor characteristics (localization, tumor size, grading), clinical and pathological TNM classification, and occurrence of lymph node metastases. Information on type and extent of neck dissection was also collected. Smoking and alcohol consumption were recorded as binary variables based on documented patient history at the time of diagnosis (yes/no). Data processing and descriptive statistical analyses were performed using Microsoft Excel 2019 (Microsoft Corporation, Redmond, WA, USA) and SPSS Statistics Version 25 (IBM Corp., Armonk, NY, USA). Continuous variables were summarized using means and standard deviations, while categorical variables were described using absolute numbers and percentages. Group comparisons were performed using the chi-square test or Student’s *t*-test, depending on the scale and distribution of the variables. A two-sided *p*-value < 0.05 was considered statistically significant. To identify independent predictors of cervical lymph node metastasis (pN > 0), multivariate logistic regression analysis was performed using JASP (JASP Team, Version 0.19.3). Variables were selected for inclusion in the multivariate model based on clinical relevance. The number of predictor variables was limited according to the number of outcome events to ensure statistical stability of the model. Only patients with complete clinical, radiological, and histopathological datasets were included in the final analysis. In cases where individual pathological parameters were missing in the original reports, the corresponding variable was recorded as unknown (e.g., pTx, cNx, Gx), while cases lacking essential core variables were excluded from analysis. Therefore, no data imputation methods were required, and analyses were performed using complete-case datasets.

During the study period, the TNM classification system for head and neck cancer was revised from the 7th to the 8th edition in 2017 [9,10]. To ensure consistency of tumor staging across the entire study period, all cases were classified according to the 7th edition of the TNM classification. Reclassification according to the 8th edition was not feasible for all cases, as key parameters introduced in the updated classification, such as depth of invasion (DOI), were not consistently documented in earlier pathological reports. Therefore, uniform application of the 7th edition was considered methodologically more appropriate to maintain comparability within the study cohort.

The primary outcome of this study was the occurrence of cervical lymph node metastasis, defined as histopathologically confirmed nodal involvement (pN > 0). Secondary outcomes included the occurrence of occult metastases (cN0/pN+) and contralateral lymph node metastases.

The main analyses regarding occult metastases, tumor site–specific metastasis patterns, number needed to treat, and diagnostic accuracy of preoperative staging were restricted to primary OSCC cases.

### 2.2. Preoperative Staging

Preoperative staging included a combination of clinical, radiological, and endoscopic examinations to determine tumor stage and nodal status. Tumor classification was performed in accordance with the TNM classification and Union for International Cancer Control (UICC) staging system. Both clinical (cTNM) and histopathological (pTNM) staging results were recorded for all patients. All patients underwent surgical neck dissection, allowing for histopathological examination of cervical lymph nodes in all cases.

Standard preoperative assessment included clinical examination, contrast-enhanced computed tomography (CT) of the head and neck, ultrasound of the cervical lymph nodes, and panendoscopy. Clinical nodal status (cN) was determined based on established radiological criteria for metastatic lymph node involvement, including increased lymph node size, round morphology, central necrosis, loss of fatty hilum, irregular borders, and suspicious contrast enhancement patterns. Final cN classification was based on the combined clinical and radiological assessment.

In patients with clinically advanced-stage tumors (cT3–cT4), additional CT imaging of the chest and abdomen was performed according to institutional staging protocols.

To evaluate the diagnostic performance of preoperative staging, clinical nodal status (cN) was compared with histopathological nodal findings (pN), which served as the reference standard. Based on this comparison, sensitivity, specificity, positive predictive value (PPV), negative predictive value (NPV), as well as false positive and false negative rates were calculated. Sensitivity was calculated as TP/(TP + FN), specificity as TN/(TN + FP), positive predictive value (PPV) as TP/(TP + FP), and negative predictive value (NPV) as TN/(TN + FN), where TP = true positive, TN = true negative, FP = false positive, and FN = false negative findings.

### 2.3. Surgical Procedure

Standard surgical management consisted of tumor resection followed by reconstruction using either local, regional, or microvascular flap techniques and cervical neck dissection (ND). The extent of ND was determined based on preoperative staging findings and intraoperative assessment. During the study period, elective neck dissection was routinely recommended for surgically treated OSCC cases at our institution, including maxillary and palatal carcinomas. Final treatment decisions were made in an interdisciplinary tumor board considering tumor extent, anatomical subsite, radiological findings, and patient-specific risk factors.

Neck dissections were performed as selective, modified radical, or radical procedures according to the classification proposed by Robbins et al. [11]. Selective ND typically included lymph node levels I–III and was the most frequently performed procedure in elective cases. Depending on tumor characteristics and clinical staging, ND was performed either unilaterally or bilaterally.

A substantial proportion of patients in this cohort underwent bilateral elective ND (reported in detail in the Results section), enabling systematic histopathological assessment of both ipsilateral and contralateral cervical lymph nodes. This approach allowed for a detailed evaluation of contralateral metastasis patterns and provided comprehensive staging information.

## 3. Results

### 3.1. Study Population

Between January 2013 and December 2019, a total of 356 patients with head and neck cancer underwent surgical treatment including neck dissection (ND) at the Department of Oral and Maxillofacial Surgery, Hannover Medical School. Of these, 287 patients with histologically confirmed oral squamous cell carcinoma (OSCC) met the inclusion criteria and were included in the final analysis, comprising 236 primary tumor cases.

### 3.2. Extent of ND

Among patients with primary OSCC, 91.9% underwent neck dissection. Bilateral selective ND according to Robbins et al. [11] was performed in 82.2% of patients and represented the most common surgical approach. In 74.2% of these cases (*n* = 144), bilateral selective ND was performed electively. Radical and modified radical neck dissections were performed less frequently (Table 1).

Among the 236 primary tumor cases, 148 patients (62.7%) were staged as clinically node-negative (cN0). Of these, 25 patients were found to have histopathologically confirmed lymph node metastases (pN+), corresponding to an occult metastasis rate of 16.9%. Only two of these patients exhibited contralateral occult metastases. This resulted in a number needed to treat (NNT) of 6 for ipsilateral elective ND and 74 for contralateral elective ND (2/148; 1.35%, 95% CI 0.16–4.80%) (Table 2).

### 3.3. Epidemiology

The 287 tumor cases included in this study were identified in 262 individual patients. This difference reflects the inclusion of patients with recurrent tumors and second primary malignancies. Among the tumor cases, 236 were primary tumors (82.2%), 43 were recurrent tumors (15.0%), and 8 were secondary tumors (2.8%).

The mean patient age at diagnosis was 64 ± 12.4 years. The study population comprised 145 male patients (55.3%) and 117 female patients (44.7%). Statistically significant sex-related differences were observed regarding tumor localization and distribution of risk factors. Primary tumors of the floor of the mouth were more frequently observed in male patients (*p* = 0.0002), whereas maxillary tumors occurred more frequently in female patients (*p* = 0.0038). Tobacco and/or alcohol use was significantly more common in male patients than in female patients (78.6% vs. 48.7%, *p* < 0.009).

Clinical follow-up examinations were routinely scheduled for up to five years after treatment. However, reliable documentation of survival status was available in only 10.1% of patients, precluding meaningful survival analysis.

### 3.4. Pattern of Lymphatic Metastases

Lymphatic metastases were assessed based on histopathological nodal status (pN). The influence of tumor site, clinicopathological parameters, and risk factors on the occurrence of cervical lymph node metastases was analyzed.

### 3.5. Tumor Site

Tumor localization significantly influenced the occurrence of cervical lymph node metastases. OSCC of the maxilla demonstrated a significantly lower risk of cervical metastasis (*p* = 0.026) and occult cervical metastasis (*p* = 0.041) compared to other tumor sites (Table 2, univariate analysis). No occult metastases were observed among clinically node-negative maxillary OSCC cases (0/18; 0%, 95% CI approximately 0–18.5%).

Overall, occult metastases were detected in 16.9% of patients with primary tumors. Occult metastases occurred exclusively in primary tumors. In patients with secondary tumors, no significant differences were observed between tumor sites and occurrence of lymph node metastases. Contralateral lymphatic metastases were observed in 14 patients, of whom 13 presented with bilateral nodal involvement. Ten of these patients had primary tumors. Only two cases of contralateral occult metastases were identified, both associated with tumors crossing the midline. Although both contralateral occult metastases occurred in midline-crossing tumors, statistical significance was not reached, likely due to limited sample size. Multivariate analysis confirmed comparable risks for lymph node metastases in primary and secondary tumors (Table 3).

### 3.6. Risk Factors

Tobacco and alcohol consumption were common risk factors among patients with OSCC. Tobacco use was reported in 49.6% of male patients and 34.2% of female patients. Combined tobacco and alcohol use was documented in 27.6% of male patients and 13.7% of female patients.

Alcohol consumption showed a non-significant trend toward increased risk of lymph node metastasis (OR 1.54, 95% CI 0.86–2.75, *p* = 0.144). In subgroup analysis, non-smokers with small tumors (T1–T2) showed a higher occurrence of lymph node metastases compared with smokers; however, this difference did not reach statistical significance (OR 1.88, 95% CI 0.80–4.41, *p* = 0.14).

In multivariate analysis, smoking remained significantly associated with nodal status (*p* = 0.011) (Table 3). This direction of association may partly reflect the subgroup observation that patients without documented risk factors and with small tumors (T1–T2) showed a comparatively higher rate of lymph node metastases in this cohort.

### 3.7. TNM Classification and Tumor Characteristics

**Figure 1:**
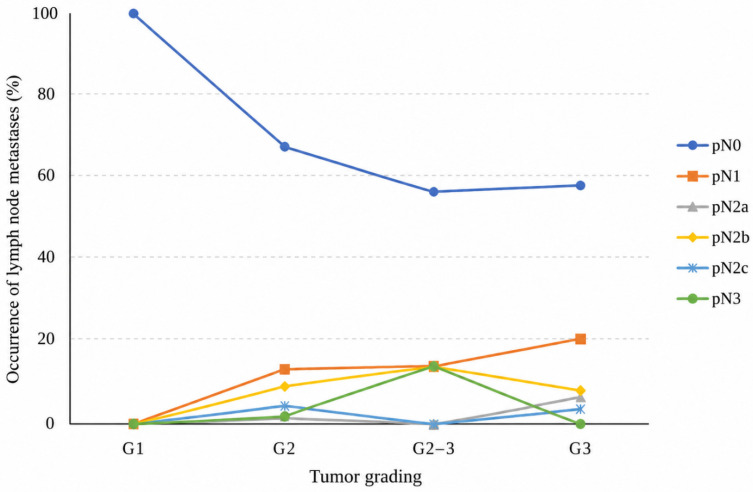
Correlation between tumor grading and the occurrence of lymph node metastases (pN).

The distribution of pTNM stages is presented in Table 4. Tumor size (pT) showed no statistically significant association with lymph node metastasis; however, a trend toward increased lymphatic involvement was observed in larger tumors (T3–T4). Histopathological grading demonstrated a significant association with lymph node metastases, with a significantly lower risk observed in G1 tumors (*p* = 0.0092) (Figure 1). Distribution of tumor sites among G1 tumors showed no evidence of site-related bias. Clinical nodal stage (cN) and histopathological grading (G stage) were identified as significant predictors of lymph node metastases in multivariate analysis (Table 3). Perineural invasion and lymphatic vessel invasion showed no statistically significant association with lymph node metastases. Distant metastases were identified in four patients.

### 3.8. Quality of Preoperative Staging Examinations

To evaluate the diagnostic performance of preoperative staging, clinical nodal status (cN) was compared with histopathological findings (pN), which served as the reference standard.

A characteristic feature of this cohort was the high rate of bilateral elective neck dissection (ND), performed in 91.9% of primary tumor cases. Among these, 82.2% underwent bilateral selective ND (*n* = 194). Of the primary tumor cases, 148 patients (62.7%) were staged as clinically node-negative (cN0), whereas 70 patients were staged as clinically node-positive (cN+).

Among patients staged as cN0, lymph node metastases were detected histopathologically in 25 cases, corresponding to a false negative rate of 0.31 and a negative predictive value (NPV) of 0.83. The false positive rate was 0.20, with a positive predictive value (PPV) of 0.65 (Table 5).

In cases of recurrent disease (*n* = 51), the false positive rate was 0.40, while no occult metastases were observed, resulting in a false negative rate of 0.0. Overall sensitivity of preoperative staging was 0.70, with a specificity of 0.80. Extended staging using CT of the chest and abdomen detected distant metastases in four patients, corresponding to a number needed to screen (NNS) of 72.

**Table 5:** Diagnostic performance of preoperative staging.

Parameter	Value
Sensitivity	0.70
Specificity	0.80
Positive Predictive Value (PPV)	0.65
Negative Predictive Value (NPV)	0.83
False Positive Rate	0.20
False Negative Rate	0.31

## 4. Discussion

This study evaluated the pattern of cervical lymph node metastases in patients with OSCC, with particular emphasis on tumor site, diagnostic staging accuracy, and the therapeutic relevance of elective ipsilateral and contralateral neck dissection. The present results demonstrate significant differences in the occurrence of lymph node metastases depending on tumor localization and histopathological grading. In particular, OSCC of the maxilla showed a significantly lower rate of cervical and occult lymph node metastases compared with other tumor sites. Notably, no occult metastases were observed in patients with maxillary OSCC in the present cohort.

Tobacco and alcohol consumption are well-established risk factors for the development of OSCC [12,13]. In the present cohort, patients without documented risk factors and with small primary tumors (T1–T2) demonstrated a relatively higher rate of lymph node metastases. This observation is consistent with clinical experience and may reflect biological heterogeneity among OSCC cases arising in patients without classical risk factors; however, this finding should be interpreted cautiously.

Multivariate analysis identified clinical nodal status (cN) and smoking as significant predictors of cervical lymph node metastases. The difference between univariate and multivariate results for smoking may reflect confounding effects of other clinicopathological variables included in the regression model, particularly clinical nodal status and tumor grading. Adjustment for these variables strengthened the observed association between smoking and nodal metastases. The observed association between smoking and nodal status should be interpreted in the context of the subgroup distribution within this cohort and does not necessarily imply a protective effect of smoking. This finding should be interpreted cautiously due to potential confounding effects.

No occult metastases were detected in cases of recurrent disease, which may be explained by previous surgical treatment of the cervical lymph nodes in these patients. A previous study demonstrated a significant influence of tumor site on overall survival, which has been attributed to differences in tumor stage distribution and resection status [14]. The reduced rate of lymph node metastases in maxillary OSCC observed in the present study supports these findings (univariate analysis). Recent treatment guidelines acknowledge a lower tendency for lymph node metastases in maxillary OSCC but do not recommend omitting elective neck dissection in these cases [3].

### 4.1. Elective Neck Dissection

In the present study, occult lymph node metastases were detected in 16.9% of primary tumor cases, with only two cases involving contralateral lymph nodes. These findings are consistent with previously reported rates in comparable patient cohorts [15,16,17]. The presence of lymph node metastases is well known to be associated with more advanced disease and reduced overall survival [16]. The high proportion of bilateral neck dissections reflects institutional surgical practice during the study period and may have introduced treatment-selection bias. At the same time, it provided the opportunity for systematic bilateral histopathological assessment of cervical lymph nodes and more reliable detection of contralateral occult metastases.

The therapeutic value of ipsilateral elective neck dissection (ND) is well established and supported by randomized prospective data demonstrating improved disease-free and overall survival following elective ND compared to therapeutic ND [4]. In the present cohort, the calculated number needed to treat (NNT) for ipsilateral elective ND was 6, further supporting its clinical relevance. However, no occult metastases were observed in patients with maxillary OSCC in this cohort. Due to the limited number of clinically node-negative maxillary cases, this subgroup analysis should be interpreted cautiously. The absence of observed occult metastases represents a clinically relevant finding but does not exclude a low underlying risk with sufficient statistical certainty.

Only two contralateral occult metastases were observed among clinically node-negative patients (2/148; 1.35%, 95% CI 0.16–4.80%). Despite the low absolute number of events, systematic bilateral histopathological assessment was available in a large proportion of the cohort. The observed event rate therefore provides meaningful evidence that contralateral occult metastases are uncommon in patients without midline-crossing tumors and supports a selective rather than routine use of contralateral elective neck dissection. These findings are consistent with current clinical practice and treatment recommendations, where contralateral elective ND is typically reserved for selected cases [18].

Postoperative morbidity and potential impairment of quality of life following ND should be considered when determining the indication for bilateral procedures. Therefore, careful patient selection remains essential, particularly in cases without clear risk factors for contralateral metastases.

### 4.2. Potential of Sentinel Lymph Node Biopsy

Sentinel lymph node biopsy (SLNB) represents a potential alternative approach for nodal staging in selected cases [19,20]. The reliability of SLNB in oral squamous cell carcinoma has been demonstrated in several prospective studies and meta-analyses. A European multicenter trial including 415 clinically cN0 patients reported a detection rate of 99.5% and a sensitivity of 86% [21], while another study including 134 patients reported detection rates of 93% and a sensitivity of 91% [22]. Meta-analyses have demonstrated overall detection rates of approximately 95% and sensitivities ranging from 86% to 94% [20,23,24,25]. Potential benefits of SLNB include reduced postoperative morbidity compared with elective ND [26,27]. SLNB has been incorporated into national diagnostic recommendations, including the guidelines of the German Society for Nuclear Medicine [28]. In many OSCC cases requiring microvascular reconstruction, transcervical access is necessary for vascular anastomosis. In such situations, the additional surgical burden of performing neck dissection may be relatively limited. Nevertheless, current evidence does not support routine replacement of elective ND by SLNB in all patients [3]. In the present cohort, the relatively high rate of occult metastases and the limited reliability of preoperative staging underline the continued importance of surgical nodal assessment, while alternative strategies such as SLNB may be considered in carefully selected cases.

### 4.3. Staging Accuracy

The high proportion of bilateral elective ND in this cohort enabled comprehensive histopathological evaluation of cervical lymph nodes and allowed for reliable assessment of preoperative staging accuracy. The observed rate of occult metastases (16.9%) and the negative predictive value of 0.83 indicate that preoperative imaging alone is insufficient to reliably exclude nodal disease. These findings are consistent with previously published data [8].

The comparatively lower positive predictive value (0.65) suggests that primary extension of neck dissection solely based on suspected nodal involvement may result in overtreatment. In this context, a stepwise surgical strategy including elective ND with intraoperative frozen section analysis may represent a pragmatic approach for optimizing surgical management.

Beyond conventional imaging, several emerging approaches have recently been proposed for preoperative prediction of occult cervical metastases in OSCC. These include radiomics-based analyses, machine learning models based on advanced imaging data, and combinations of inflammatory biomarkers with clinical and radiological parameters. Recent studies reported promising predictive performance, particularly in early-stage (T1–T2/cN0) disease, potentially improving risk stratification and reducing unnecessary elective neck dissections. Nevertheless, external validation and prospective multicenter studies are still required before these approaches can be incorporated into routine preoperative decision-making [29,30].

### 4.4. Limitations

This study has several limitations that should be considered when interpreting the results. First, the retrospective single-center design may introduce selection bias and limit the generalizability of the findings to other clinical settings.

Second, reclassification according to TNM 8th edition was not feasible for all cases because depth of invasion (DOI) was not consistently documented in earlier pathological reports. As DOI represents an important prognostic factor in contemporary OSCC staging, omission of DOI may have reduced the precision of risk stratification, particularly in early-stage tumors. Third, reliable survival and regional control data were not available for the entire cohort and therefore could not be analyzed. Consequently, conclusions regarding lymph node management are based on observed patterns of nodal metastasis and diagnostic accuracy rather than direct oncologic outcome measures. Recurrent and second primary tumors were included for descriptive characterization of the overall cohort but were not used as the basis for the main conclusions regarding elective neck dissection in primary OSCC. Furthermore, subgroup analysis of maxillary OSCC included a comparatively limited number of cases, which may reduce statistical power and limit the interpretation of subgroup-specific findings.

Despite these limitations, the study benefits from a relatively large cohort and a high proportion of bilateral elective neck dissections, allowing for a comprehensive histopathological evaluation of cervical lymph nodes and reliable assessment of staging accuracy.

## 5. Conclusions

Elective ipsilateral neck dissection remains an essential component in the surgical management of oral squamous cell carcinoma, supported by the considerable rate of occult metastases and the limited reliability of preoperative staging examinations observed in this cohort. In contrast, the benefit of contralateral elective neck dissection appears limited in patients without midline-crossing tumors, as reflected by the low rate of contralateral occult metastases and the high number needed to treat. Furthermore, maxillary OSCC and well-differentiated (G1) tumors demonstrated a significantly lower risk of lymph node metastases. These findings support a more individualized risk-adapted approach to elective neck dissection in selected low-risk cases.

## Figures and Tables

**Table 1:** Type of Neck Dissections.

*n*	Ipsilateral	Contralateral
194	SND	SND
16	SND	none
3	none	SND
1	SND	(M)RND
18	(M)RND	SND
4	(M)RND	(M)RND
236		

SND = selective neck dissection, (M)RND = (modified) radical neck dissection.

**Table 2:** Occult metastases according to tumor site.

	Total Cases	pN+	cN0	Occult Metastases (cN0/pN+)
Tumor Site	*n*	(*n*, %)	(*n*, %)	(*n*, %)
Anterior	57	18 (31.6%)	43 (75.4%)	10 (23.3%)
Tongue	56	23 (41.1%)	35 (62.5%)	9 (25.7%)
Mandible	41	17 (41.5%)	20 (48.7%)	3 (15.0%)
Maxilla/palate	30	5 (16.7%)	18 (60.0%)	0 (0%)
Tongue base	19	7 (36.8%)	11 (57.9%)	0 (0%)
Retromolar triangle	18	7 (38.9%)	12 (66.7%)	2 (16.7%)
Planum buccale	12	5 (41.7%)	6 (50.0%)	1 (16.7%)
Lip	3	0 (0%)	3 (100%)	0 (0%)
total	236	82	148	25

**Table 3:** Multivariate binary logistic regression analysis of predictors for cervical lymph node metastases (pN > 0) in OSCC.

Variable	OR	95% CI	*p*
cT	0.96	0.74–1.25	0.772
cN	1.57	1.28–1.92	<0.001
G	2.54	1.10–5.86	0.030
alcohol	1.54	0.86–2.75	0.144
primary vs. secondary	0.54	0.23–1.28	0.162
smoking	0.37	0.17–0.80	0.011

cT and G were entered as ordinal variables. Reference categories were defined as cN0, non-smoker, and primary tumor.

**Table 4:** pTNM classification of included patients.

		N	%
		287	100
Tumor size (pT)			
	pT1	95	33.1
	pT2	86	29.96
	pT3	34	11.85
	pT4a	53	18.47
	pT4b	3	1.05
	pTx	16	5.57
Lymph node metastases (pN)			
	pN0	176	61.33
	pN1	36	12.54
	pN2a	6	2.09
	pN2b	26	9.06
	pN2c	12	4.18
	pN3	7	2.44
	pNx	24	8.36
Distant metastases (M)			
	M0	283	98.6
	M1	4	1.4
Grading (G)			
	G1	14	4.9
	G2	182	63.4
	G2-3	7	2.4
	G3	24	8.4
	Gx	60	20.9
Lymph infiltration (L)			
	L0	113	39.4
	L1	13	4.5
	Lx	161	56.1
Nerve invasion (Pn)			
	Pn0	102	35.54
	Pn1	21	7.32
	Pnx	164	57.14
Vein invasion (V)			
	V0	120	41.8
	V1	4	1.4
	Vx	163	56.8
Extracapsular spread (ecs)			
	+	17	5.9
	−	270	94.1
Resection state (R)			
	R0	240	83.6
	R1	18	6.3
	Rx	29	10.1

## Data Availability

The datasets used and/or analyzed during the current study are available from the corresponding author on reasonable request.
